# F216. SLEEP QUALITY AND CLINICAL IMPROVEMENT IN FIRST EPISODE PSYCHOSIS

**DOI:** 10.1093/schbul/sby017.747

**Published:** 2018-04-01

**Authors:** L Prayosha Villa, Lyndsay Schmidt, Tyler M Moore, Monica Calkins, Christian G Kohler

**Affiliations:** 1 University of Pennsylvania; 2 University of Pennsylvania School of Medicine

## Abstract

**Background:**

Sleep disturbance is a common feature in early psychosis. Sleep quality has shown to be associated with both symptom severity and clinical improvement in persons with chronic illness.

Understanding the influence of sleep quality in early psychosis can be beneficial in determining interventions for coordinated specialty care (CSC). Using patients from a CSC intervention program for first episode psychosis, we investigated the association between subjective sleep quality with clinical response and clinical symptom correlates.

**Methods:**

Participants were consecutive patients admitted between March 2015 to June 2017 who underwent coordinated specialty care at Penn PERC (Psychosis Evaluation and Recovery Center). Eligible participants were young persons ages 16–35 years who had experienced onset of psychosis within 3 years prior to intake and who underwent 2-years of CSC for early psychosis, including cognitive therapy for psychosis recovery (CT-R), medication management, family education and occupational support. Standardized self and observer based rating scales evaluating sleep quality (PSQI) and other clinical symptoms, e.g., anxiety (BAI), depression (BDI) and affective states (PANAS), and clinical improvement (CGI-I) were administered at intake, after 3 months, 6 months and subsequently every 6 months of CSC. Participants provided informed consent. 48 participants completed assessment at 2-time points between intake and 2–4 months later and 38 underwent assessment at 3 time points, including 6–7 months following intake. Correlational analyses were performed on PSQI change (slope) over 3 assessments and change in BAI, BDI, PANAS-negative, PANAS-positive. Analysis were further stratified by improvement – CGI-I <2 (much improvement) (n=17) and CGI >3 (little/no improvement) (n=21).

**Results:**

Of 48 patients, average age at intake was 22 years (Male:Female=40:8; Caucasian:African-American/Other=28:20). Primary analyses of sleep quality and clinical improvement included participants with three PSQI rating timepoints over 6–7 months of CSC (n=38). Overall PSQI ratings did not change significantly over time. BAI and BDI-II scores significantly decreased over time, indicating subjective clinical improvement with treatment. There was a trend for positive correlations among PSQI, and BAI and BDI-II scores. When stratified by improvement, those rated ‘much improved’ group greater reduction of PSQI scores.

**Discussion:**

We found that improved sleep quality was present in participants who experienced much global clinical improvement over 6 months of CSC. In addition, better sleep quality correlated with reduced depression and anxiety symptoms. Though these findings do not address direction of causality, our findings indicate that improving sleep quality should be a specific focus in treatment of early psychosis. Further analysis will be conducted to investigate the relationship between sleep and clinical improvement using other clinical measures, such as symptom severity, and the dataset will be expanded to include data through the end of 2017.

